# Early signaling, referral, and treatment of adolescent chronic pain: a study protocol

**DOI:** 10.1186/1471-2431-12-66

**Published:** 2012-06-11

**Authors:** Jessica S Voerman, Sylvia Remerie, L Esther de Graaf, Petra van de Looij-Jansen, Tessa Westendorp, Ina van Elderen, Frouwkje de Waart, Jan Passchier, Anke Dommisse van Berkel, Cora de Klerk

**Affiliations:** 1 Department of Medical Psychology and Psychotherapy, Erasmus MC University Medical Hospital, PO Box 2040, Rotterdam, CA, 3000, the Netherlands; 2 Rijndam rehabilitation centre, PO Box 23181, Rotterdam, KD, 3001, the Netherlands; 3 Municipal Public Health Services Rotterdam-Rijnmond, PO Box 70032, Rotterdam, LP, 3000, the Netherlands; 4 Centre for Youth and Family Rijnmond, PO Box 3074, Rotterdam, AB, 3003, the Netherlands; 5 Faculty of Psychology and Education, Department of Clinical Psychology, VU University Amsterdam, Van der Boechorstraat 1, Amsterdam, BT, 1081, the Netherlands

**Keywords:** Chronic pain, Adolescents, Signaling, Referral, Treatment, Internet intervention

## Abstract

**Background:**

Chronic pain is prevalent among young people and negatively influences their quality of life. Furthermore, chronic pain in adolescence may persist into adulthood. Therefore, it is important early on to promote the self-management skills of adolescents with chronic pain by improving signaling, referral, and treatment of these youngsters. In this study protocol we describe the designs of two complementary studies: a signaling study and an intervention study.

**Methods and design:**

The signaling study evaluates the Pain Barometer, a self-assessed signaling instrument for chronic pain in adolescents. To evaluate the feasibility of the Pain Barometer, the experiences of youth-health care nurses will be evaluated in semi-structured interviews. Also, we will explore the frequencies of referral per health-care provider. The intervention study evaluates Move It Now, a guided self-help intervention via the Internet for teenagers with chronic pain. This intervention uses cognitive behavioural techniques, including relaxation exercises and positive thinking. The objective of the intervention is to improve the ability of adolescents to cope with pain. The efficacy of Move It Now will be examined in a randomized controlled trial, in which 60 adolescents will be randomly assigned to an experimental condition or a waiting list control condition.

**Discussion:**

If the Pain Barometer is proven to be feasible and Move It Now appears to be efficacious, a health care pathway can be created to provide the best tailored treatment promptly to adolescents with chronic pain. Move It Now can be easily implemented throughout the Netherlands, as the intervention is Internet based.

**Trial registration:**

Dutch Trial Register NTR1926

## Background

In 1986 the International Association for the Study of Pain (IASP) formulated the following definition of pain: ‘Pain is an unpleasant sensory and emotional experience associated with actual or potential tissue damage, or described in terms of such damage. Note: pain is always subjective. Each individual learns the application of the word through experiences related to injury in early life’. Most child studies define chronic pain as pain that persists continuously or intermittently for at least three months [1-6]. In the general population approximately 25% of children and adolescents (0–18 years) suffer from chronic pain [4]. Limb pain, headache, and abdominal pain are the most reported types of pain. A prevalent combination is headache plus abdominal pain [7]. The prevalence of chronic pain is the highest in girls aged 12 to 14 years [4]. In 30% of the children and adolescents pain seems to persist after two years [5]. Additionally, 59% of the women and 39% of the men who reported pain in childhood also reported pain in early adulthood [8].

Chronic pain negatively influences the quality of life of adolescents [9]. Children and adolescents with pain are often unable to meet friends and to pursue hobbies [7]. Additionally, they may report disturbed sleep, loss of appetite, frequent use of medication, subclinical depression, and school absence. School absenteeism is also burdensome for parents, who often have to take days off from work [10]. Chronic pain also significantly impacts on the health care system [11]. Approximately 43% of children and adolescents with chronic pain utilize health care and 53% use pain medication at least once every three months. The frequency of health care use is positively related to age, pain intensity, and pain duration [6].

Some studies suggest that parents influence the way children and adolescents experience pain. Pain seems to be less intense when parents distract their child from pain, while pain appears to be worse when parents focus on the child’s pain [12]. Additionally, parents of children and adolescents with chronic pain often have chronic pain themselves [13]. Pain in adolescents also seems to be predicted by depressive symptoms, somatic complaints (other than pain), reduced leisure time activities, number of friends, and recent parental divorce [14]. Additionally, chronic pain in adolescents is related to coping [15]. Adolescents with an avoidant coping style and adolescents with a dependent coping style, report the most disabled functioning. They have higher levels of depression and anxiety than other adolescents with chronic pain.

In summary, chronic pain in adolescents is influenced by many factors. This is in accordance with the biopsychosocial model [16], which states that pain is caused by a complex interaction between biological (e.g., genetics), psychological (e.g., attention), and social variables (e.g., role models). Differences in the interaction between these variables may explain differences in pain expression between pain patients. For this reason, different types of treatment, i.e. medical and psychological, might be useful for adolescents with chronic pain.

### Signaling and referral

To optimally treat adolescents with chronic pain it is important to signal pain complaints at an early stage. The Pain Questionnaire was the first instrument that was developed for indexing pain and pain parameters in children and adolescents, irrespective of pain localization [4]. The questionnaire consists of questions about the experience, location, frequency, duration, and intensity of pain. The Pain Questionnaire was designed for usage in the general population but is not considered suitable as a signaling instrument as it does not allow for the impact of pain. This means that currently chronic pain is not taken into account in the regular signaling and registration practice of the Preventive Youth Health Care in the Netherlands. To signal chronic pain in the general population a short signaling questionnaire should be developed. Additionally, a referral protocol is needed to offer the most tailored treatment to adolescents with chronic pain.

### Psychological treatment

A systematic review of randomized controlled trials (RCTs) has shown that cognitive-behavioral therapy (CBT) and relaxation exercises are effective in reducing pain frequency and pain intensity in children and adolescents [17]. CBT may be effective in providing strategies that help adolescents to cope with their pain. As a result, pain-related symptoms of anxiety and depression may also be reduced and quality of life may improve. However, only few studies have examined if psychological treatment also enhances adolescents’ well-being [18,19]. The results of a study by Trautmann and colleagues [18] showed that their self-help training program improved pain catastrophizing, but not depression and quality of life.

More studies on the effect of psychological treatment on coping and well-being are needed. Unfortunately, inclusion is often problematic in face-to-face cognitive behavioral therapy [19]. Adolescents have difficulty combining vis-à-vis therapy sessions with homework and activities with friends. Additionally, youngsters with bodily symptoms are often reluctant to consult a psychologist. Psychological interventions using an Internet format could therefore be more attractive to adolescents. Advantages of internet therapy are that it is available for all geographical districts and adolescents can work through the program at any place and any time.

### Internet interventions

Stinson and colleagues concluded in their review that Internet-based self-management interventions seems to improve health outcomes in children and adolescents with various health conditions, including pain [20]. In 2005, Hicks and colleagues tested the efficacy of an Internet-supported treatment for adolescents with chronic pain in the general population [19]. One month after treatment the adolescents in the experimental group experienced significantly reduced pain compared to adolescents in the waiting list group. More recently, Palermo and colleagues (2009) evaluated an Internet-delivered family CBT intervention [21]. Results showed that pain-related disability and pain intensity were reduced more in the Internet group than in the waiting list group. These effects were maintained at three months follow-up. In summary, these findings suggest that Internet interventions may be a promising alternative to face-to-face therapy in teenagers with chronic pain.

### Current study

In the current manuscript we describe the study protocols of two related studies: a referral study and an intervention study. The main aim of these studies is to improve signaling, referral, and treatment of adolescents with chronic pain. By collaborating with various (mental) health-care institutions, a health-care pathway can be created, through which adolescents can receive the best tailored treatment. Firstly, a signaling instrument for chronic pain was developed. The instrument includes a referral protocol for implementation in the preventive health examinations of the Public Health Services Rotterdam. Secondly, a guided self-help intervention via the Internet was developed for adolescents with chronic pain. The intervention includes cognitive behavioural techniques and relaxation exercises and is one of the referral options in the referral protocol. The primary objective of this intervention is to improve the way adolescents cope with pain.

Our main research questions are: 1. What is the feasibility of the signaling instrument and the accompanying referral protocol in the context of Preventive Youth Health Care? 2. What is the efficacy of the guided self-help intervention via the Internet?

## Methods referral study

### Population

Each year, the Municipal Public Health Services in Rotterdam preventively examine health conditions and developmental problems in approximately 12.000 first-grade adolescents from several secondary schools in the Rotterdam area. The signaling and referral instrument developed for this study will be part of this preventive examination. The instrument was filled out by approximately 6.000 adolescents in the period between October 2008 and July 2011. The Medical Ethical Committee of Erasmus MC University Medical Hospital, Rotterdam, The Netherlands approved the study protocol (MEC-2009-195).

### The pain barometer

The Pain Barometer is a self-assessed signaling and referral instrument for chronic pain in adolescents aged 12 to 17 years. The instrument is based on the Pain Questionnaire [4], literature research, and expert knowledge (e.g., a medical doctor and researcher from Rijndam rehabilitation centre, psychologists from the Erasmus MC University Medical Hospital and researchers, nurses, and a medical doctor from the Preventive Youth Health Care in Rotterdam). The Pain Barometer consists of 12 questions about several characteristics of pain (e.g., duration, localization, frequency, intensity, and consequences). Chronic pain is defined as pain that lasts for at least three months, recurrently or continuously.

Also part of the Pain Barometer is a referral protocol that states several referral options and their criteria for adolescents with chronic pain. The referral options are presented in Table 1. Additionally, adaptations were made to a digital dossier, e.g. Electronic Child Dossier (ECD), to enable registration. The newly developed registration categories are presented in Table 2.

**Table 1:** The Pain Barometer referral protocol

**Referral option**	**Criteria**
No referral for pain	the adolescent does not experience the pain as a problem or other problems are more urgent
General practitioner	the adolescent and/or the parents worry about a medical cause of the pain or the youth nurse suspects a medical cause of the pain
School doctor	the adolescent is absent from school for more than two days per month or the adolescent regularly misses gym lessons at school or the school nurse is not sure if it is necessary to refer to the general practitioner
Move It Now	the adolescent is aged 12 to 17 years old and there is no apparent medical cause of pain according to the general practitioner and there are no serious psychosocial problems and the adolescent withdraws from activities or becomes overburdened
Youth rehabilitation	there is no apparent medical cause of pain and the adolescents’ pain is located in the limbs or back and the adolescent experiences pain as a big problem and Move It Now does not seem to be sufficient

**Table 2:** The Pain Barometer registration system

**Registration categories**	**Answer categories**
Conversation details	Free text
Chronic pain, but other problems more important	Yes No
Referral to the school doctor	Yes No
Referral to the GP	Yes No
Referral to Youth rehabilitation	Yes No
Referral to Move It Now	Yes No

### Procedure

When the adolescent has filled out the Pain Barometer, a computer calculates whether he or she has chronic pain. Adolescents who are in chronic pain discuss their pain complaints with a youth health-care nurse during the standard consultation procedure with the Public Health Services. The youth health-care nurse uses the referral protocol to determine which treatment option is the best. He or she registers details about the consultation and the referral in the ECD.

To evaluate the feasibility of the Pain Barometer and the referral protocol, the experiences of the youth health-care nurses are evaluated in semi-structured interviews by telephone. Examples of questions are ‘Can you describe the adolescents you referred to the school doctor, General Practitioner (GP), rehabilitation center, or Move It Now?’ and ‘Were the referral options clear?’

### Qualitative analyses

The interviews will be audio-recorded and transcribed. The transcripts will be analyzed using the program ATLAS.ti [22]. First, unique themes will be identified. Second, the unique themes will be clustered according to main themes.

### Quantitative analyses

The ECD will be used to determine the frequencies of referral per health service, i.e. school doctor, GP, rehabilitation centre, or Move It Now.

## Methods intervention study

### Population

The study sample will consist of 60 adolescents. Adolescents are eligible to participate if they meet the following criteria: age 12 to 17 years; chronic pain for at least three months; abdominal pain, headache, back pain or limb pain; no apparent medical cause of pain (an exception is made for adolescents with migraine); withdrawing from activities or feeling overburdened; access to the Internet at home; fluent in the Dutch language; no current psychological treatment for pain; no severe psychosocial problems.

Recruitment of participants takes place via the referral study, the Internet, the media, and rehabilitation centers and hospitals in the Netherlands. The study started April 2009. The inclusion of patients will end in June 2012. The three-month follow-up period will be finished in September 2012.

### Intervention

Move It Now is a translated and adapted version of the intervention program developed by Hicks and colleagues [19]. It is a guided self-help intervention via Internet for adolescents aged 12 to 17 years with chronic pain. The objective of the intervention is to improve the way adolescents cope with pain. The intervention is based on the principles of cognitive-behavioral therapy. Adolescents independently work through seven online modules. The modules aim to teach important life skills, such as problem solving and relaxation. Additionally, the adolescents learn to change dysfunctional thoughts and beliefs. Other topics include deep breathing and improving self-esteem and interpersonal relationships. All topics in the modules are presented in Table 3. Additionally, a therapist from the Rijndam rehabilitation centre contacts the participants by e-mail or telephone each week. This therapeutic contact consists of emotional support and a discussion about the online modules and exercises. Parents work through two online modules and have contact with the therapist three times (at the beginning, in the middle, and at the end of the intervention). In the parent modules, information is given about how parents should handle the pain of their child. Parents are strongly advised to encourage their child to complete Move It Now.

**Table 3:** Overview of Move It Now modules

**Module number**	**Module subject**	**Module contents**
1	Background and goals	Psycho-education about pain and its consequences/treatment goals
2	Pain killers and breathing	Strategies to relieve pain /deep breathing
3	Relaxation	Advantages of relaxation/two relaxation exercises, one for the whole body and one in which fantasy is used
4	Thinking and feeling	Strategies to reduce negative thinking patterns
5	Helpful thinking	Reasoning errors/ helpful thoughts
6	Staying active	Staying active physically (e.g., sports) and socially (e.g., going out with friends)/a short relaxation exercise, which can be used to relax rather quickly
7	Making a plan	Developing a plan about what to do when in pain at home or at school

Much attention has been given to the design and lay-out of the Move It Now website. For example, the adolescents are led through the online chapters by an animated female guide using a voice-over. Furthermore, several interactive elements were developed to lead the adolescents to the material that fits their response, i.e. tailoring. For example, if the adolescent reports having headaches, only information about headaches will appear.

### Instruments

The measures in the intervention study are in accordance with the recommendations of the Pediatric Initiative on Methods, Measurement and Pain Assessment in Clinical Trials (PedIMMPACT) [23].

### Demographic data

Demographic data collected includes the adolescents’ gender, date of birth, ethnicity, and educational level and was gathered using a self-assessed questionnaire.

### The pain diary

The participating adolescents keep a daily diary for one week. Pain intensity is measured by several multiple choice questions and a Visual Analogue Scale (VAS), with no pain and worst pain ever at the respective ends. Prior research has shown that the reliability and validity of this scale is high [24].

### Pain coping questionnaire

Pain coping is measured by the Pain Coping Questionnaire (PCQ) [25]. The PCQ consists of 39 items, which are evaluated on a five point scale (with 1 = never and 5 = very often). The PCQ has three dimensions: approach, problem-focused avoidance, and emotion-focused avoidance and eight subscales: information seeking, problem solving, seeking social support, positive self-statements, behavioral distraction, cognitive distraction, externalizing, and internalizing/catastrophizing. Higher scores indicate greater use of the coping strategy. The Dutch version of the PCQ has good psychometric properties [26].

### Pediatric migraine disability assessment scale

Pain-related disability is measured with an adapted version of the Pediatric Migraine Disability Assessment Scale (PedMIDAS) [27,28]. The PedMIDAS consists of 6 items. Participants report the number of days a specific aspect of functioning was impaired in the previous three months due to pain. A higher score on the PedMIDAS is indicative of more pain-related disability. The reliability and validity of the PedMIDAS are acceptable [27].

### Sleep

The following self-assessed questions are used to measure sleeping problems: Do you have sleeping problems?, What do you think causes your sleeping problems?, and How many hours of sleep do you get in a period of 24 hours?.

### Child health questionnaire

The Child Health Questionnaire (CHQ) [29] is used to measure physical and psychosocial wellbeing of adolescents. The CHQ consist of 87 items, divided over 10 multi-item subscales and two single item questions: physical functioning, role functioning emotional, role functioning behavioral, role functioning physical, bodily pain, general behavior, mental health, self esteem, general health perceptions, change in health, family activities, and family cohesion. Higher scores indicate better functioning and well-being. The psychometric properties of the Dutch version of the CHQ are good, even when completed via the Internet [30,31].

### EQ-5D-5L developed by the EuroQol group

The EQ-5D-5L developed by the EuroQol Group is also used to measure adolescents’ health-related quality of life. The EQ-5D-5L consists of 5 dimensions: mobility, self-care, daily activities, pain/discomfort, and depression/anxiety. Each dimension comprises 5 levels: no problems, slight problems, moderate problems, major problems, and extreme problems. A higher score on the EQ-5D-5L dimensions is indicative of a worse quality of life. The psychometric qualities of the 5 level version of the EQ-5D seem promising [32].

### Illness behaviour encouragement scale

The Illness Behaviour Encouragement Scale (IBES) [33] measures the rewarding of pain behavior by parents. The IBES consists of 12 items, which are evaluated on a five point scale (with 0 = never and 4 = always). The items of the IBES comprise two subscales: rewarding by parents in a pain situation and rewarding by parents in a pain-free situation. Higher scores indicate more rewarding of pain by parents. The reliability and validity of the IBES is satisfactory [34].

### Pain catastrophizing scale – child version

Pain catastrophizing is measured by the Pain Catastrophizing Scale Child version (PCS-C) [35], which consists of 13 items. Items are scored on a five point scale (with 0 = not at all and 4 = extremely). Higher scores indicate higher levels of pain catastrophizing. Research has shown that the Dutch version of the PCS-C is reliable and has satisfactory predictive validity [35-37].

## Design

**Figure 1:**
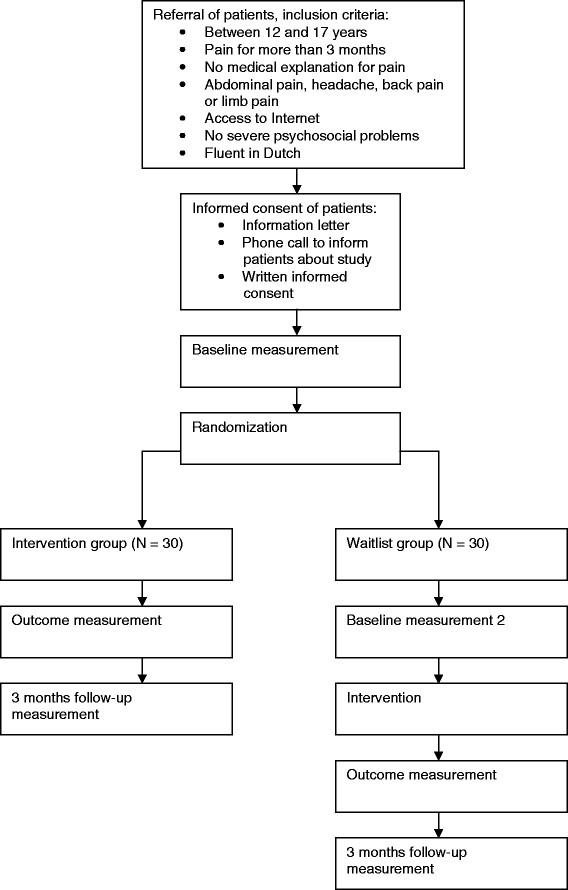
Design of the randomized controlled trial.

The intervention study is a randomized controlled trial. Participants are automatically randomly assigned to the experimental group or the waiting list group by the Move It Now website. The Move It Now website uses a randomization list which has been generated by an independent statistician. Block randomization has been used to keep the group sizes equal. Prior to entry of the participants into the study, both the researchers and the therapist are blinded for randomization. Figure 1 shows the design and the anticipated flow of participants.

Adolescents in the waiting list group wait seven weeks before they are allowed to start with the intervention. Adolescents in the experimental group fill out the same online questionnaires and the online Pain Diary three times in total: at baseline, directly after the intervention, and at three months follow-up. Adolescents in the waiting list group fill out the same online questionnaires and the online Pain Diary four times in total: at baseline, after the waiting period of seven weeks, directly after the intervention, and at three months follow-up. The Medical Ethical Committee of Erasmus MC University Medical Hospital, Rotterdam, The Netherlands has approved the study protocol (MEC-2009-195). The study has been registered at the Netherlands Trial Register, which is part of the Dutch Cochrane Centre (NTRcode 1926).

### Procedure

Adolescents are informed about Move It Now by their youth health-care nurse or their medical specialist. When an adolescent decides to participate in Move It Now, he or she contacts the researcher by mail or e-mail. Thereafter, the researcher contacts the adolescent and one of the parents by telephone. In this phone call, the researcher checks the inclusion criteria, gives information, and asks the adolescent and the parent to send their signed informed consent forms.

When the researcher has received the signed informed consent forms, the adolescent and the parent are registered at the Move It Now website. An automatic e-mail with their login codes is sent to them. When the adolescent logs into the website, he or she first fills out the online questionnaires; the PedMIDAS, the self-constructed sleep questionnaire, the CHQ, the PCQ, the IBES, and the PCS-C. For logistic reasons, the EQ-5D-5L and a separate reply form are sent to the adolescent by e-mail. Additionally, the adolescent keeps an online Pain Dairy for one week. After this baseline measurement, the adolescent and the parent receive an automatic e-mail that informs them whether they can start with Move It Now directly in the intervention group or if they have to wait for seven weeks.

### Statistical analyses

Power calculations have shown that for an effect of d = .70 on the questionnaires, a sample size of 25 participants per group is needed (power 80%, alpha 0.05). Adjusting for potential study withdrawal (20%), we estimate that 60 participants should be included.

Multi-level modelling will be applied for longitudinal analysis. This analysis allows handling incomplete data with a minimal loss of information. Results of the measurements at baseline, post-treatment, and at three months follow-up will be presented. Linear splines will be used to test the differences in outcomes between time points. For all differences, effect sizes will be reported, with d = .2-.5 indicating a weak effect, d = .5-.8 indicating a medium effect and d = > .8 indicating a strong effect [38].

The EQ-5D-5L, in combination with the CHQ [29], will be used for the economic evaluation of Move It Now. The results will be expressed as cost per Quality Adjusted Life Year (QALY).

## Discussion

The primary aim of our study is to improve signaling, referral, and treatment of adolescents with chronic pain. In the referral study we will explore if the Pain Barometer, the signaling and referral instrument, is feasible in the context of preventive Youth Health-Care. In the intervention study we will explore if Move It Now, the guided self-help program via Internet, is efficacious.

Our study is the first in developing an instrument to signal chronic pain in adolescents that directly can be applied in practice. We expect the Pain Barometer to be a feasible instrument. A limitation of the referral study is that it is not possible to explore the validity of the Pain Barometer in this setting.

Move It Now is one the first tailored and guided self-help interventions via the Internet for adolescents with chronic pain. We expect the participants to cope with their pain more actively directly after the Move It Now intervention. We also hypothesize that Move It Now reduces pain frequency, pain duration, pain intensity, and pain disability. A strength of the intervention study is that it includes important but often neglected, outcome variables, i.e. coping with pain and quality of life. Additionally, the intervention is not only directed at the adolescents, but also at the parents. A limitation of the intervention study is that there will be no control group at follow-up, since the intervention is offered to the adolescents after a short waiting period. Furthermore, a waiting list group is used as a control. Therefore it will not be clear if the specific or nonspecific factors, such as attention, influence the intervention.

If the Pain Barometer appears to be feasible, it can be used to signal and refer adolescents with chronic pain at an early stage in various health care settings. If Move It Now is proved to be efficacious it can be easily implemented throughout the Netherlands, as the intervention is internet based. Then it will also be possible to perform a large scale (cost-)effectiveness study to examine the effect of Move It Now in daily practice. Additionally, future research should examine which elements are working for which adolescents. The Move It Now intervention could also easily be adapted to suit the needs of adolescents with other chronic health issues.

## Abbreviation

CBT, Cognitive-behavioral therapy; CHQ, Child health questionnaire; ECD, Electronic child dossier; EQ-5D-5L, EQ-5D-5L developed by the EuroQol Group; IASP, International association for the study of pain; IBES, Illness behaviour encouragement scale; PedIMMPACT, Pediatric initiative on methods, measurement and pain assessment in clinical trials; PedMIDAS, Pediatric migraine disability assessment scale; PCQ, Pain coping questionnaire; PCS-C, Pain catastrophizing scale-child version; RCT, Randomized controlled trial; SDQ, Strength and difficulties questionnaire; QALY, Quality adjusted life year; VAS, Visual analogue scale.

## Competing interests

The authors declare that they have no competing interests.

## Authors’ contributions

JSV LEdG ADvB PvdLJ IvE carried out the studies. JSV CdK drafted the first version of the manuscript. CdK JP SR TW FdW substantially contributed to conception and design of the study. All authors read and corrected draft versions. They approved the final version of this manuscript.

## Pre-publication history

The pre-publication history for this paper can be accessed here:

http://www.biomedcentral.com/1471-2431/12/66/prepub

## References

[B1] Lynch AM Kashikar-Zuck S Goldschneider KR Jones BA Psychosocial risks for disability in children with chronic back pain J Pain 2006 7 4 244 251 10.1016/j.jpain.2005.11.001 16618468

[B2] Scharff L Langan N Rotter N Scott-Sutherland J Schenck C Tayor N McDonald-Nolan L Masek B Psychological, behavioral, and family characteristics of pediatric patients with chronic pain: a 1-year retrospective study and cluster analysis Clin J Pain 2005 21 5 432 438 10.1097/01.ajp.0000130160.40974.f5 16093749

[B3] Lynch AM Kashikar-Zuck S Goldschneider KR Jones BA Sex and age differences in coping styles among children with chronic pain J Pain Symptom Manage 2007 33 2 208 216 10.1016/j.jpainsymman.2006.07.014 17280926

[B4] Perquin CW Hazebroek-Kampschreur AA Hunfeld JA Bohnen AM van Suijlekom-Smit LW Passchier J van der Wouden JC Pain in children and adolescents: a common experience Pain 2000 87 1 51 58 10.1016/S0304-3959(00)00269-4 10863045

[B5] Perquin CW Hunfeld JA Hazebroek-Kampschreur AA van Suijlekom-Smit LW Passchier J Koes BW van der Wouden JC The natural course of chronic benign pain in childhood and adolescence: a two-year population-based follow-up study Eur J Pain 2003 7 6 551 559 10.1016/S1090-3801(03)00060-0 14575668

[B6] Roth-Isigkeit A Thyen U Stoven H Schwarzenberger J Schmucker P Pain among children and adolescents: restrictions in daily living and triggering factors Pediatrics 2005 115 2 e152 e162 10.1542/peds.2004-0682 15687423

[B7] Haraldstad K Sorum R Eide H Natvig GK Helseth S Pain in children and adolescents: prevalence, impact on daily life, and parents' perception, a school survey Scand J Caring Sci 2011 25 1 27 36 10.1111/j.1471-6712.2010.00785.x 20409061

[B8] Brattberg G Do pain problems in young school children persist into early adulthood? A 13-year follow-up Eur J Pain 2004 8 3 187 199 10.1016/j.ejpain.2003.08.001 15109969

[B9] Hunfeld JA Perquin CW Duivenvoorden HJ Hazebroek-Kampschreur AA Passchier J van Suijlekom-Smit LW van der Wouden JC Chronic pain and its impact on quality of life in adolescents and their families J Pediatr Psychol 2001 26 3 145 153 10.1093/jpepsy/26.3.145 11259516

[B10] Schechter N Finley GA, McGrath PJ, Chambers CT Treatment of acute and chronic pain in the outpatient setting. Bringing Pain Relief to Children: Treatment Approaches 2006 Humana Press Inc, New Jersey

[B11] Perquin CW Hunfeld JA Hazebroek-Kampschreur AA van Suijlekom-Smit LW Passchier J Koes BW van der Wouden JC Insights in the use of health care services in chronic benign pain in childhood and adolescence Pain 2001 94 2 205 213 10.1016/S0304-3959(01)00355-4 11690734

[B12] Walker LS Williams SE Smith CA Garber J Van Slyke DA Lipani TA Parent attention versus distraction: impact on symptom complaints by children with and without chronic functional abdominal pain Pain 2006 122 1–2 43 52 16495006 10.1016/j.pain.2005.12.020 PMC3232036

[B13] Laurell K Larsson B Eeg-Olofsson O Headache in schoolchildren: association with other pain, family history and psychosocial factors Pain 2005 119 1–3 150 158 16298064 10.1016/j.pain.2005.09.030

[B14] Larsson B Sund AM Emotional/behavioural, social correlates and one-year predictors of frequent pains among early adolescents: influences of pain characteristics Eur J Pain 2007 11 1 57 65 10.1016/j.ejpain.2005.12.014 16480907

[B15] Claar RL Baber KF Simons LE Logan DE Walker LS Pain coping profiles in adolescents with chronic pain Pain 2008 140 2 368 375 10.1016/j.pain.2008.09.007 18938032 PMC3100727

[B16] Turk DC Flor H Gatchel RJ, Turk DC Chronic pain: a biobehavioral perspective Psychosocial factors in pain 1999 Guilford Press, New York

[B17] Eccleston C Morley S Williams A Yorke L Mastroyannopoulou K Systematic review of randomised controlled trials of psychological therapy for chronic pain in children and adolescents, with a subset meta-analysis of pain relief Pain 2002 99 1–2 157 165 12237193 10.1016/s0304-3959(02)00072-6

[B18] Trautmann E Kroner-Herwig B A randomized controlled trial of Internet-based self-help training for recurrent headache in childhood and adolescence Behav Res Ther 2010 48 1 28 37 10.1016/j.brat.2009.09.004 19782343

[B19] Hicks CL von Baeyer CL McGrath PJ Online psychological treatment for pediatric recurrent pain: a randomized evaluation J Pediatr Psychol 2006 31 7 724 736 16093516 10.1093/jpepsy/jsj065

[B20] Stinson J Wilson R Gill N Yamada J Holt J A systematic review of internet-based self-management interventions for youth with health conditions J Pediatr Psychol 2009 34 5 495 510 10.1093/jpepsy/jsn115 19029142

[B21] Palermo TM Wilson AC Peters M Lewandowski A Somhegyi H Randomized controlled trial of an Internet-delivered family cognitive-behavioral therapy intervention for children and adolescents with chronic pain Pain 2009 146 1–2 205 213 19695776 10.1016/j.pain.2009.07.034 PMC2760656

[B22] Scientific Software Development GmbH ATLAS.ti: The Knowledge Workbench 1997 Scientific Software Development, Berlin

[B23] McGrath PJ Walco GA Turk DC Dworkin RH Brown MT Davidson K Eccleston C Finley GA Goldschneider K Haverkos L Core outcome domains and measures for pediatric acute and chronic/recurrent pain clinical trials: PedIMMPACT recommendations J Pain 2008 9 9 771 783 10.1016/j.jpain.2008.04.007 18562251

[B24] Jensen MP Karoly P Braver S The measurement of clinical pain intensity: a comparison of six methods Pain 1986 27 1 117 126 10.1016/0304-3959(86)90228-9 3785962

[B25] Reid GJ Gilbert CA McGrath PJ The Pain Coping Questionnaire: preliminary validation Pain 1998 76 1–2 83 96 9696461 10.1016/s0304-3959(98)00029-3

[B26] Bandell-Hoekstra IE Abu-Saad HH Passchier J Frederiks CM Feron FJ Knipschild P Coping and Quality of Life in relation to headache in Dutch schoolchildren Eur J Pain 2002 6 4 315 321 10.1053/eujp.2002.0343 12161097

[B27] Hershey AD Powers SW Vockell AL LeCates S Kabbouche MA Maynard MK PedMIDAS: development of a questionnaire to assess disability of migraines in children Neurology 2001 57 11 2034 2039 10.1212/WNL.57.11.2034 11739822

[B28] Weel S Merlijn V Passchier J Koes B van der Wouden J van Suijlekom-Smit L Hunfeld J Development and psychometric properties of a pain-related problem list for adolescents (PPL) Patient Educ Couns 2005 58 2 209 215 10.1016/j.pec.2004.08.014 16009299

[B29] Landgraf JM Abetz L Ware JE The CHQ User's Manual 1996 The Health Institute, New England Medical Centre, Boston

[B30] Raat H Landgraf JM Bonsel GJ Gemke RJ Essink-Bot ML Reliability and validity of the child health questionnaire-child form (CHQ-CF87) in a Dutch adolescent population Qual Life Res 2002 11 6 575 581 10.1023/A:1016393311799 12206578

[B31] Raat H Mangunkusumo RT Landgraf JM Kloek G Brug J Feasibility, reliability, and validity of adolescent health status measurement by the Child Health Questionnaire Child Form (CHQ-CF): internet administration compared with the standard paper version Qual Life Res 2007 16 4 675 685 10.1007/s11136-006-9157-1 17286197 PMC1832149

[B32] Herdman M Gudex C Lloyd A Janssen M Kind P Parkin D Bonsel G Badia X Development and preliminary testing of the new five-level version of EQ-5D (EQ-5D-5L) Qual Life Res 2011 20 10 1727 1736 10.1007/s11136-011-9903-x 21479777 PMC3220807

[B33] Walker LS Zeman JL Parental response to child illness behavior J Pediatr Psychol 1992 17 1 49 71 10.1093/jpepsy/17.1.49 1545321

[B34] Bijttebier P Vertommen H Antecedents, concomitants, and consequences of pediatric headache: confirmatory construct validation of two parent-report scales J Behav Med 1999 22 5 437 456 10.1023/A:1018605423614 10586381

[B35] Crombez G Bijttebier P Eccleston C Mascagni T Mertens G Goubert L Verstraeten K The child version of the pain catastrophizing scale (PCS-C): a preliminary validation Pain 2003 104 3 639 646 10.1016/S0304-3959(03)00121-0 12927636

[B36] Mascagni T Bijttebier P Crombez G Vlaeyen J De pain catastrophizing scale for children: eerste psychometrische bevindingen Gedragstherapie 2001 34 325 336

[B37] Verstraeten K Bijttebier P Crombez G Mertens G Mascagni T Goubert L De pain catastrophizing scale for children: predictieve validiteit in een klinische steekproef Gedragstherapie 2003 36 299 307

[B38] Cohen J Statistical power analysis for the behavioral sciences 1988 Lawrence Erlbaum, Hillsdale, New York

